# Importance of Humanities and Compassionate Care in the Age of Artificial Intelligence

**DOI:** 10.31729/jnma.v63i2091.9224

**Published:** 2025-11-30

**Authors:** Madhusudan Subedi

**Affiliations:** 1 Patan Academy of Health Sciences, Lagankhel, Lalitpur, Nepal

**Keywords:** *Artificial intelligence*, *compassionate care*, *empathy*, *health humanities*, *trust*

## Abstract

The increasing integration of artificial intelligence into healthcare presents both opportunities and challenges. Health science education has long prioritized biomedical knowledge and technical skills, emphasizing diagnosis, procedures, and evidence-based practice. Although essential, humanistic dimensions of care continue to be marginalized in curricular design. Integrating health humanities enhances diagnostic sensitivity and emotional intelligence. Health education programs combining ethics and humanities foster moral reasoning and professional identity, reinforcing values like integrity and accountability. Creative activities like writing, art, theater, and storytelling help students reflect on clinical experiences, observe better, and grow emotionally. As AI transforms clinical practice, the ability to understand and respond to the human experience of illness remains vital. Embedding health humanities into professional training ensures ethically grounded and culturally sensitive care. The integration of AI into healthcare should be guided by medical humanities scholars to ensure that empathy remains central and technology complements compassionate care.

## INTRODUCTION

Empathy, compassion, and trust are core principles of patient-centered healthcare.^1^ However, the growing use of artificial intelligence (AI) in healthcare offers new possibilities and challenges. AI is being used to analyze patient data and assist in diagnosis, and healthcare professionals must convey empathy and compassion in their interactions with patients.^2^ Healthcare professionals who have empathy training will be more effective in communicating AI-generated recommendations to patients, thereby strengthening trust and promoting adherence to treatment plans.^3^ By integrating AI with empathetic clinical practice, there will be improvements in both health outcomes and patient satisfaction.^1^

This short communication highlights the importance of a humanities and compassionate care curriculum in health science education and practice. It emphasizes the growing need to integrate the humanities into their curricula to foster empathy, ethical practices, and patient-centered care. Drawing on students’ feedback from medical humanities courses conducted in other countries and Nepal, the paper illustrates how such initiatives will enhance students’ understanding of human experiences in health, illness, disease, disability, suffering, and caring. Finally, the editorial proposes strategic ways forward to embed health humanities more deeply within health education systems.

## MEDICAL EDUCATION SYSTEM

Health science education has traditionally focused on biomedical knowledge and technological skills, with a strong emphasis on diagnosis, procedures, and evidence-based practice. While this scientific foundation is essential, it has often overshadowed the humanistic aspects of care. Disciplines such as literature, philosophy, ethics, history, anthropology, sociology, visual arts, and film offer valuable perspectives on the human experience of illness, disease, disability, ageing, and dying.^1-3^ Clinical data alone cannot provide these human experience components. Health humanities nurture empathy, critical reflection, cultural sensitivity, and ethical awareness, all of which are vital for compassionate and patient-centered care.^4^ However, despite ongoing discussions about the need for reform in curriculum, humanities continue to occupy a marginal and often symbolic place in health science curricula. Neglecting the humanities components limits the holistic growth of future health professionals.^3^

Efforts to integrate empathy and patient-centered communication into medical education are a new phenomenon in South Asia.^5^ However, in the West, there is a relatively long history of inclusion of the humanities in medical education. Research has shown that medical university students with a humanities and science background perform better in practice than those with a science background alone.^6^ In their curriculum, universities have included the disciplines of art, drama, sociology, anthropology, history, philosophy, law, religious studies, and visual arts in their Medical, Nursing, and Allied Health Sciences curriculum.^5,6^

Beginning in the 21st century, the integration of medical humanities has played a significant role in shaping medical education reform worldwide.^2,6^ Medical education is becoming more learner-focused to develop professional values and to encourage students to take a holistic view of the patients’ experiences.

## IMPORTANCE OF HUMANITIES IN HEALTH SCIENCE CURRICULUM

Humanities play a vital role in health science education by fostering empathy, ethical awareness, cultural competence, and resilience among future health professionals.^5^ Engaging with literature, philosophy, and arts helps students connect with narratives of illness, suffering, and healing, encouraging them to view patients as whole individuals rather than clinical cases. Exposure to the humanities enhances diagnostic skills by training learners to observe and interpret with greater sensitivity, while also developing emotional intelligence^1^ Ethical reasoning is another area where the humanities offer essential support. Medical decisions often involve complex moral dilemmas that cannot be resolved through scientific knowledge alone. Humanities provide frameworks for navigating these challenges, promoting respect for patient rights and the cultivation of professional conscience.^2-4^

In increasingly diverse societies, cultural competence has become a key requirement for effective healthcare. Disciplines like anthropology, sociology, and history help students understand the social and cultural contexts of health beliefs and practices, enabling more inclusive and responsive care.^5^ Integrating cultural perspectives into biomedical training can improve decision-making and reduce systemic barriers. Humanities offer reflective and expressive tools to help practitioners process grief, uncertainty, and moral distress.^6^ Similarly, art-based approaches not only deepen empathy but also support the well-being of health professionals, making the humanities a valuable resource for preventing burnout.^1^

Despite these benefits, the humanities remain marginal in many health science curricula. Studies show that exposure is often limited, fragmented, and treated as optional rather than essential. Institutional barriers such as time constraints, a heavy focus on biomedical content, and a lack of consensus on teaching methods continue to hinder integration.^7^ However, promising developments are emerging in various countries. These examples suggest that with institutional commitment, the humanities can be effectively integrated into health science education, enriching both learning and practice.

## INTEGRATION OF MEDICAL HUMANITIES IN HEALTH SCIENCE CURRICULUM

Integrating medical humanities into health science education requires more than occasional electives or symbolic gestures; it calls for structured, longitudinal inclusion across the curriculum. Rather than treating humanities as optional supplements, they should be embedded as essential components that enrich clinical training and professional development.^7^ The importance of fostering dialogue between healthcare management and the humanities to challenge the dominance of quantifiable metrics and reorient healthcare around patient experience. This integration can take diverse forms.

Integrating literature, philosophy, and history into health education helps students understand human experience, ethical reasoning, and cultural perspectives. These subjects, when designed for medical learners, enrich their capacity for empathy and critical thinking. Intercalated humanities content in basic sciences in medicine and nursing offer a structured way to explore these disciplines within medical training.^7^

Humanistic themes can also be woven into clinical teaching, such as by analyzing patient narratives alongside case histories, encouraging students to reflect on the emotional and social dimensions of illness. Longitudinal tracks that combine humanities and ethics help cultivate moral reasoning and shape professional identity over time, reinforcing the values of empathy, integrity, and accountability. Creative expression through reflective writing, visual arts, and theater offers students meaningful ways to process clinical experiences, build observational skills, and sustain emotional resilience.^5-8^ These approaches do not compromise scientific rigor; rather, they broaden the epistemological base of health sciences to include both analytical precision and humanistic understanding. Such integration affirms that caring for patients involves not only technical expertise but also ethical sensitivity, cultural awareness, and emotional depth. Engaging in narrative medicine fosters self-awareness and reflective practice by encouraging health professionals to examine both their own experiences and those of their patients. This process of meaning-making significantly enhances the psychological resilience and overall well-being of both health professionals and patients.^7^

## MEDICAL HUMANITIES IN NEPAL

In 2018 and 2021, Patan Academy of Health Sciences (PAHS) introduced medical humanities for undergraduate medical and nursing students, respectively, to cultivate empathy, ethical sensitivity, and a deeper understanding of human experiences related to illness, disability, ageing, and death. The PAHS expanded the humanities in public health in 2025. The study findings indicated that students perceived the Medical Humanities course as a meaningful and enjoyable component of their medical education, fostering the development of empathy and compassion.^8^ Another study provides evidence of the impact of a medical humanities course on increasing medical students’ empathy scores.^9^ In light of global concerns regarding declining empathy in healthcare, these results underscore the importance of integrating and expanding such courses within Nepal’s medical curriculum.

This initiative aligns with global trends in medical education that emphasize the importance of integrating arts, literature, and social sciences to enhance clinical and public health practice. The impact of the medical humanities course revealed positive student feedback and a measurable increase in empathy scores.^8,9^ Findings indicated that students appreciated the course for its role in enhancing their emotional intelligence and interpersonal skills. The Medical Education Commission may initiate a draft of a health humanities curriculum to ensure the curriculum reflects national priorities, including economic constraints, healthcare access, rapid socioeconomic transitions, rising education levels, and the impact of cultural and ethnic diversity on health.^10^

## INTEGRATING HUMANITIES INTO HEALTH PROFESSIONAL EDUCATION: A STRATEGIC FRAMEWORK

To foster ethically grounded, empathetic, and culturally sensitive healthcare in the digital age, health humanities should be systematically embedded within health professional education and training. This integration must:

- Cultivate humanistic competencies: Embed health humanities modules that nurture empathy, ethical reasoning, and compassionate care alongside clinical and technological proficiency.
- Contextualize locally: Align curricular content with country-specific health systems and sociocultural realities, ensuring relevance and responsiveness to local needs.
- Promote interdisciplinary innovation: Encourage interdisciplinary research and teaching that integrates health humanities, artificial intelligence, and clinical practice. Ensure that technological progress supports and enhances compassionate, human-centered care rather than substituting it.
- Enable global knowledge exchange: Facilitate crosscountry collaboration to synthesize evidence, share innovative practices, and evaluate outcomes that strengthen people-centered health systems.
- Engage in regional and international dialogue: Participate in global fora to exchange lessons and promote compassionate, culturally attuned, and ethically sound healthcare practices.
- Collaborate for global advocacy: Partner with international organizations to advance a global initiative that integrates health humanities into health professional training, balancing empathy with digital competence.
- Monitor and align with system goals: Establish mechanisms to track progress, linking humanities integration with broader goals of health system strengthening and universal health coverage.
- Develop a collaborative model: Train healthcare professionals to use AI responsively and empathically, and prioritize human dignity in every technological innovation.

## CONCLUSION

In the age of artificial intelligence, the role of health humanities has become more urgent and indispensable. As health science education increasingly embraces technological innovation, there is a real risk of neglecting the human dimensions of care. Humanities offer essential tools for cultivating empathy, ethical reasoning, cultural competence, and emotional resilience. These qualities cannot be replicated by algorithms or derived from biomedical training alone. Although challenges such as curricular overload and epistemological bias persist, successful models of integration show that meaningful reform is both possible and necessary. The question is no longer whether the humanities have a place in health science education, but how they can be embedded in ways that are sustainable, contextually relevant, and institutionally supported. As artificial intelligence reshapes clinical practice, the ability to understand and respond to the human experience of illness remains central to compassionate care. Health humanities should be an essential foundation. Its role is critical to shaping ethically grounded, contextually sensitive approaches to health and technology. Artifical Intelligence is becoming more integrated into clinical and consumer health settings, enhancing the technological role of medicine. Medical and health humanities scholars should play an important role in guiding how AI can be integrated with empathy and compassionate care.
